# Genome-wide patterns of noncoding and protein-coding sequence variation in the major fungal pathogen *Aspergillus fumigatus*

**DOI:** 10.1093/g3journal/jkae091

**Published:** 2024-05-02

**Authors:** Alec Brown, Jacob L Steenwyk, Antonis Rokas

**Affiliations:** Department of Biological Sciences, Vanderbilt University, Nashville, TN 37235, USA; Evolutionary Studies Initiative, Vanderbilt University, Nashville, TN 37235, USA; Department of Biological Sciences, Vanderbilt University, Nashville, TN 37235, USA; Evolutionary Studies Initiative, Vanderbilt University, Nashville, TN 37235, USA; Department of Molecular and Cell Biology, Howards Hughes Medical Institute, University of California, Berkeley, CA 94720, USA; Department of Biological Sciences, Vanderbilt University, Nashville, TN 37235, USA; Evolutionary Studies Initiative, Vanderbilt University, Nashville, TN 37235, USA

**Keywords:** fungal genomics, evolution, *Aspergillus fumigatus*, noncoding region, strain, virulence factor, selection, polymorphism, divergence, strain heterogeneity, pathobiology

## Abstract

*Aspergillus fumigatus* is a deadly fungal pathogen, responsible for >400,000 infections/year and high mortality rates. *A. fumigatus* strains exhibit variation in infection-relevant traits, including in their virulence. However, most *A. fumigatus* protein-coding genes, including those that modulate its virulence, are shared between *A. fumigatus* strains and closely related nonpathogenic relatives. We hypothesized that *A. fumigatus* genes exhibit substantial genetic variation in the noncoding regions immediately upstream to the start codons of genes, which could reflect differences in gene regulation between strains. To begin testing this hypothesis, we identified 5,812 single-copy orthologs across the genomes of 263 *A. fumigatus* strains. In general, *A. fumigatus* noncoding regions showed higher levels of sequence variation compared with their corresponding protein-coding regions. Focusing on 2,482 genes whose protein-coding sequence identity scores ranged between 75 and 99%, we identified 478 total genes with signatures of positive selection only in their noncoding regions and 65 total genes with signatures only in their protein-coding regions. Twenty-eight of the 478 noncoding regions and 5 of the 65 protein-coding regions under selection are associated with genes known to modulate *A. fumigatus* virulence. Noncoding region variation between *A. fumigatus* strains included single-nucleotide polymorphisms and insertions or deletions of at least a few nucleotides. These results show that noncoding regions of *A. fumigatus* genes harbor greater sequence variation than protein-coding regions, raising the hypothesis that this variation may contribute to *A. fumigatus* phenotypic heterogeneity.

## Introduction

Invasive aspergillosis (IA) is one of the deadliest fungal diseases for humans. IA is estimated to be responsible for over 400,000 infections per year with a mortality rate of >50% (Bongomin *et al*. 2017), with recent estimates being even higher (Denning 2024). Most IA cases (>90%) are caused by *Aspergillus fumigatus* (Steinbach *et al*. 2012; Rokas *et al*. 2020), a saprophytic fungus commonly found in the soil (Flores *et al*. 2014) as well as urban environments, such as waste piles and hospitals (Wirmann *et al*. 2018). In its natural environment, *A. fumigatus* plays an important role in nitrogen and carbon recycling (Latgé and Chamilos 2019). *A. fumigatus* has adapted over time to survive environmental pressures, such as high temperatures, variation in pH, and low oxygen availability (Bhabhra and Askew 2005; Park and Yu 2016; Rees *et al*. 2017), and to compete with other microorganisms for resources (Latgé and Chamilos 2019). Recently, the World Health Organization included *A. fumigatus* in its first ever list of fungal “priority pathogens,” a testament to its seriousness as a threat to public health (WHO 2022).

*A. fumigatus* typically reproduces via asexual spores (conidia), which are released into the air for eventual germination. While some spores eventually return to the soil, others are inhaled by humans and interact with the epithelium of the lung (Chotirmall *et al*. 2013). Aided by their small diameter (2–3 µm) and hydrophobic outer layer, these spores can subsequently reach the lung alveoli (Croft *et al*. 2016). Once in the lung, *A. fumigatus* must survive a hostile environment and host defense system (Bertuzzi *et al*. 2018). Immunocompetent individuals clear these spores, but immunocompromised ones are at risk of developing IA (Cadena *et al*. 2021).

Several species closely related to *A. fumigatus* are not considered pathogenic (de Vries *et al*. 2017; Rokas *et al*. 2020; Mead *et al*. 2021). For example, *Aspergillus fischeri* is a close relative of *A. fumigatus* (the 2 species share >90% average nucleotide sequence identity and >95% average amino acid sequence identity between orthologs), yet *A. fischeri* is less virulent and is not considered clinically relevant (Mead *et al*. 2019; Steenwyk *et al*. 2020). Early genomic comparisons between 2 strains of *A. fumigatus* (Af293 and A1163) and 1 strain of *A. fischeri* (NRRL 181) revealed a set of genes uniquely present in *A. fumigatus* (Fedorova *et al*. 2008). However, a more recent genomic examination of 18 *Aspergillus* section *Fumigati* strains, representing 13 species found that 206 known genetic determinants of virulence in *A. fumigatus* are all shared between *A. fumigatus* and at least one other closely related, nonpathogenic species (Mead *et al*. 2021). Finally, recent examinations of genomic variation between the genomes of hundreds of *A. fumigatus* isolates (Barber *et al*. 2021; Horta *et al*. 2022; Lofgren *et al*. 2022) have revealed that *A. fumigatus* has an open pangenome with ∼70% of its genes being highly conserved across strains (core) and that orthologs from both clinical and environmental strains exhibit a high degree of sequence conservation.

Variation in noncoding regions can also contribute to phenotypic variation, including gene expression variation between and within species (Carroll 2005; Hill *et al*. 2021). We have previously demonstrated that noncoding regions between 2 *A. fumigatus* reference strains, Af293 and A1163 (Brown *et al*. 2022; Colabardini *et al*. 2022), as well as between *A. fumigatus* and its nonpathogenic close relatives (Brown *et al*. 2022) are highly variable. For example, we found that 418 *A. fumigatus* genes exhibit a different rate of evolution in their noncoding regions (relative to nonpathogenic close relatives), including the noncoding regions of 25 genes that are known genetic determinants of *A. fumigatus* virulence. Examination of these noncoding regions revealed numerous single-nucleotide and insertion/deletion (indel) differences between *A. fumigatus* and closely related nonpathogenic species (Brown *et al*. 2022).

To increase our knowledge of noncoding region variation within *A. fumigatus* and how levels of noncoding sequence variation compared with levels of protein-coding sequence variation, we examined the genomes of 263 *A. fumigatus* strains (using 2 *A. fischeri* strains as an outgroup) using 2 different tests of positive selection: the McDonald–Kreitman (MK) test (McDonald and Kreitman 1991; Murga-Moreno *et al*. 2019) and the Hudson–Kreitman–Aguadé (HKA) test (Hudson *et al*. 1987; Ferretti *et al*. 2012). Examination of relative levels of sequence polymorphism to divergence of the noncoding and protein-coding regions of 2,482 genes using the MK test identified 472 noncoding and 217 protein-coding regions with signatures of positive selection, including 18 known genetic determinants of *A. fumigatus* virulence with evidence of selection in their noncoding regions. The HKA test identified 207 noncoding and 4 protein-coding regions whose polymorphism to divergence ratio differed from a neutral locus, including 4 genetic determinants of *A. fumigatus* virulence with evidence of selection in their noncoding regions. Molecular function terms enriched for genes associated with the noncoding regions that showed evidence of selection in both the MK and HKA tests, include ion binding, transcriptional regulation, and stress response. These results demonstrate that *A. fumigatus* noncoding regions are typically more variable and more often under positive selection than their protein-coding counterparts, raising the hypothesis that they, too, may contribute to phenotypic differences between *A. fumigatus* strains.

## Methods

### Genomic data collection

**Fig. 1.**
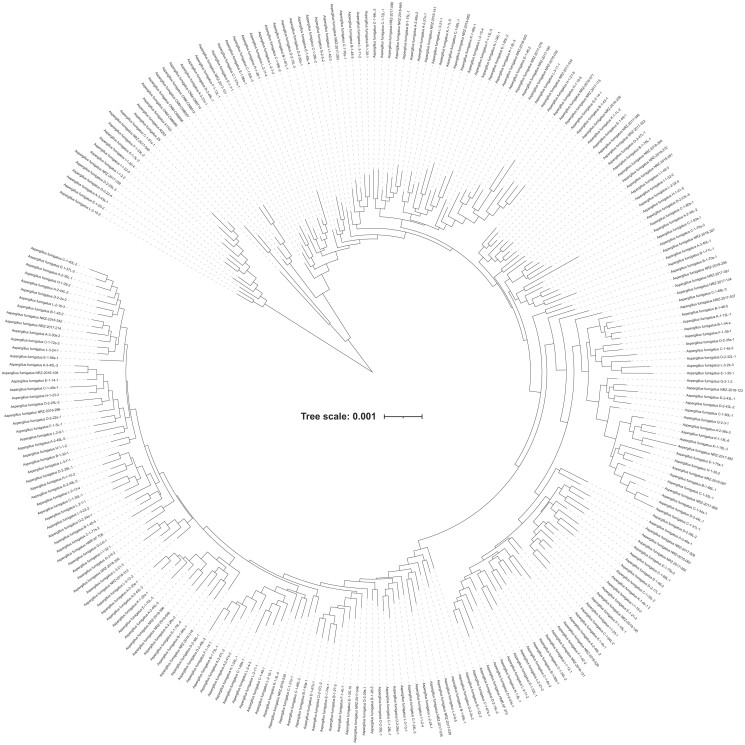
Phylogeny of the 263 *A. fumigatus* strains used in this study. A phylogenetic tree of the 263 *A. fumigatus* strains used in this study was generated by pruning from a larger *Aspergillus* species tree inferred from analyses of 1,362 protein-coding regions (Steenwyk *et al*. 2024).

All *Aspergillus* genomes are publicly available and were downloaded from NCBI (https://www.ncbi.nlm.nih.gov/); detailed information is provided in Supplementary Table 1. We used 263 *A. fumigatus* strains. Strains of *A. fumigatus* exhibit low population structure (Fig. 1), consistent with findings from other *A. fumigatus* proteomic studies (Barber *et al*. 2021; Lofgren *et al*. 2022). We used 2 *A. fischeri* strains as our outgroup because a population sample of *A. fischeri* strains was not available at the time of our study.

### Identification of single-copy orthologous genes

To infer single-copy orthologous genes across all 265 taxa, we used OrthoFinder, version 2.4.0 (Emms and Kelly 2015). OrthoFinder clustered genes into orthogroups from sequence similarity information obtained using the program DIAMOND version 2.0.9 (Buchfink *et al*. 2015) with the proteomes of the 263 *A. fumigatus* strains and 2 *A. fischeri* strains (Fig. 1). Fungal proteomes were obtained from a previously published study (Steenwyk *et al*. 2024). Key parameters used during sequence identity search include an *e*-value threshold of 1 × 10^−3^ with a percent identity cutoff of 30% and a percent match cutoff of 70%. We considered genes to be single-copy orthologs if they were within the cutoff thresholds and were present in all 265 taxa.

### Retrieval of noncoding regions

To identify highly conserved noncoding regions, we first retrieved the noncoding sequences directly upstream of the first codon of all single-copy orthologous genes from all genomes. Noncoding sequence retrieval was performed using a custom Python script, which can be found at https://github.com/alecbrown24/General_Bio_Scripts (adapted from https://github.com/shenwei356/bio_scripts). We retrieved the first 1,500 bp of noncoding sequence directly upstream of the first codon of each gene and used these sequences to generate FASTA files of noncoding regions, as well as FASTA files of single-copy orthologous protein-coding sequences using Python version 3.8.2. For some noncoding regions, there were <1,500 bp of noncoding sequence between the first codon of the gene of interest and an upstream gene; in these instances, only the intergenic region was used for subsequent analyses.

### Alignment and identification of conserved noncoding and protein-coding regions

Multiple sequence alignments for all noncoding and protein-coding regions were constructed using MAFFT, version 7.453, with default parameter settings (Katoh *et al*. 2002). Codon-based alignments were inferred from the corresponding protein sequence alignments using pal2nal, version 14 (Suyama *et al*. 2006). Sequence identity in protein-coding and noncoding regions was calculated from their corresponding multiple sequence alignment files using AliStat version 1.12 (Wong *et al*. 2020). The percent sequence identity for each position in the alignment was calculated from the fraction of sites with the same nucleotide across all taxa. Sequence identity for protein-coding and noncoding regions can be found in Supplementary Tables 2 and 3, respectively.

To measure evolutionary conservation across individual alignment sites, we implemented the PhyloP program as part of the Phylogenetic Analysis with Space/Time Models (PHAST) suite of programs (Ramani *et al*. 2019). PhyloP scores reflect the evolutionary conservation of individual nucleotide sites relative to the degree of conservation expected under neutrality. A positive score is predictive of evolutionary conservation, and a negative score is predictive of evolutionary acceleration relative to neutral expectations.

### Phylogenetic tree inference

A phylogenetic tree of the 265 strains used in this study (Fig. 1) was generated by pruning from a larger *Aspergillus* species phylogeny (Steenwyk *et al*. 2024) using the Treehouse software in R using default parameters (Steenwyk and Rokas 2019). Individual protein-coding region and noncoding region trees were inferred using IQ-TREE, version 2.0.6 (Minh *et al*. 2020), with “GTR + I + G + F” as it was the best fitting substitution model (Waddell and Steel 1997; Vinet and Zhedanov 2011).

### Identifying signatures of selection in *A. fumigatus* protein-coding and noncoding regions

We used the protein-coding and/or noncoding region alignments to calculate the fractions of polymorphic (differences between *A. fumigatus* strains) and divergent sites (differences between *A. fumigatus* and the outgroup *A. fischeri*) for nonsynonymous, synonymous, and noncoding sites using the standard MK test function as part of the iMKT software in R (Murga-Moreno *et al*. 2019).

**Fig. 2.**
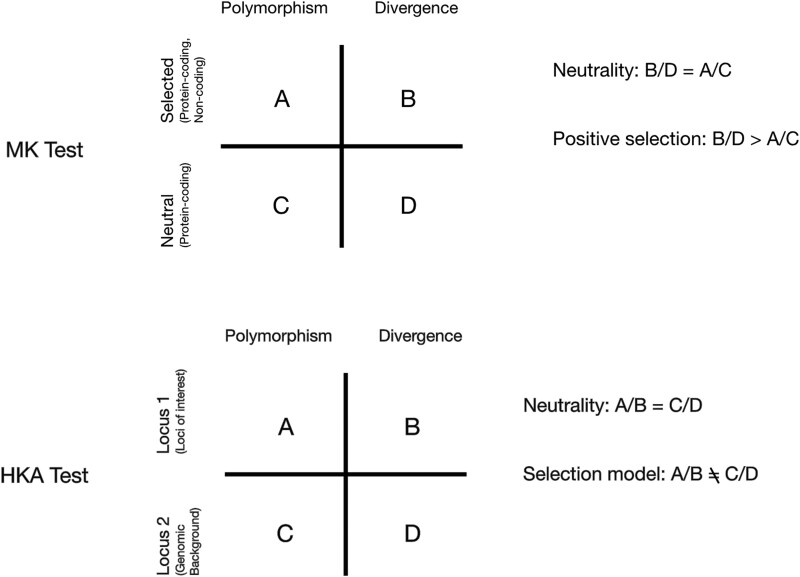
A brief overview of the MK and HKA tests of selection. The MK test (top) compares the polymorphisms (i.e. sites that vary within *A. fumigatus*) and divergence (sites that are fixed within *A. fumigatus* but differ from *A. fischeri*) between selected sites (nonsynonymous or noncoding) and neutral sites (synonymous) between the protein-coding and noncoding regions of a given gene. For protein-coding regions, nonsynonymous sites are compared with synonymous sites, while for noncoding regions, all sites are considered nonsynonymous sites and are compared with the synonymous sites of the associated protein-coding region. Under a neutral model, the ratio of selected and neutral sites that are polymorphic is the same as the ratio of selected and neutral sites that are divergent. When the ratio of divergence is greater than the ratio of polymorphism, the MK test assumes that the selection is acting to fix advantageous nonsynonymous changes, resulting in positive selection. The HKA test (bottom) compares the levels of polymorphism and divergence between 2 loci (the locus of interest and a reference, neutral locus). When the ratio of polymorphism within species is equal to the ratio of divergence between species in the 2 loci, both loci are evolving neutrally. Should these ratios differ, we conclude that selection is occurring at the locus of interest.

For protein-coding regions, the ratio of polymorphic nonsynonymous to synonymous sites was compared with the ratio of divergent nonsynonymous to synonymous sites. For noncoding regions, the ratio of polymorphic noncoding to synonymous sites was compared with the ratio of divergent noncoding to synonymous sites (Fig. 2). For each MK test, the null hypothesis (H0) assumed that the ratio of selected vs. neutral divergent sites was similar to the ratio of selected vs. neutral polymorphic sites. We compared H0 with an alternative hypothesis (H1) in which there are more divergent sites than polymorphic sites across a given protein-coding or noncoding region, indicating positive selection. To determine whether H1 was significantly different from H0 for each of the codon-based alignments, we used Fischer's exact test with a statistical significance threshold of *P* < 0.05 and a Bonferroni-adjusted alpha value <0.01 to adjust for multiple testing. Results for both protein-coding and noncoding regions can be found in Supplementary Table 4.

The HKA test was also implemented, which compares the rate of polymorphism within *A. fumigatus* to divergence (between *A. fumigatus* and *A. fischeri*) at multiple loci (Hudson *et al*. 1987; Fig. 2). The HKA test assumes that if 2 loci are evolving neutrally, the ratio of polymorphism to divergence at these loci should be relatively constant. We compared loci of interest to neutral loci using the HKADirect program (Ferretti *et al*. 2012). Neutral loci were determined by comparing each of the 2,482 loci to the genomic background (i.e. to the rest of the remaining 2,481 loci in the dataset) using Tajima D's test as part of the HKADirect program. The null hypothesis (H0) assumes that the patterns of genetic variation within a species (polymorphism) and the patterns of genetic differentiation between species (divergence) are consistent with neutral evolution. Under these conditions, the polymorphism-to-divergence ratio is similar between the loci of interest and neutral loci. We compared H0 with an alternative hypothesis (H1) in which assumes that the patterns of polymorphism and divergence at the loci of interest deviate from those of neutral loci due to the action of natural selection (Fig. 2). We used Fischer's exact test with a statistical significance threshold of *P* < 0.05 to determine significance. Results for both protein-coding and noncoding regions can be found in Supplementary Table 5.

Unlike the MK test, whose results can be used to directly compare the protein-coding and the noncoding regions of each gene, the HKA test instead compares each protein-coding and noncoding region to neutral loci. Thus, the MK test was used to determine differences in signatures of selection between noncoding regions and their associated protein-coding regions while the HKA test was used to detect signatures of selection in specific noncoding/protein-coding regions when compared with neutrally evolving noncoding/protein-coding regions.

Finally, we note that we used only 2 *A. fischeri* genomes to estimate divergence in our MK and HKA tests because a population sample of genomes of *A. fischeri* strains was not available at the time of our study. It is typical that one or a few sequences are used to estimate divergence (e.g. Xue and Bloom 2020). Furthermore, the number of polymorphisms shared between species is typically a tiny fraction of the polymorphisms found within each species; for example, a study of 16 strains of *Saccharomyces cerevisiae* and 24 strains of *Saccharomyces paradoxus* revealed that <1.5% of the single-nucleotide polymorphisms were shared between species (Elyashiv *et al*. 2010).

### Functional enrichment analyses of genes with signatures of selection

To determine whether genes with signatures of selection in either their protein-coding or noncoding regions were enriched for particular functional categories, we implemented the gene ontology (GO) tool g:PROFILER (Raudvere *et al*. 2019), using a Bonferroni correction for a significance threshold with a significance threshold of *P* < 0.05. We performed 4 separate analyses of functional enrichment among genes that significantly differed from the null hypothesis based on the MK test of protein-coding and noncoding regions as well as the HKA test of protein-coding and noncoding regions. Each of these gene sets was compared with a general background set that includes all the features/gene names in the Ensembl genome database with at least 1 GO annotation for *A. fumigatus*. All functional enrichment analyses used a *P*-value cutoff of 0.05. All genes found to be statistically significant can be found in Supplementary Table 6. We also compared our list of genes under selection in either the MK test and/or HKA test to a previously curated set of 206 genetic determinants of *A. fumigatus* virulence (Steenwyk *et al*. 2021).

### Examination and visualization of mutational signatures

To identify interesting examples of sequence variation between *A. fumigatus* strains for noncoding regions of genes of interest, we visualized and compared multiple sequence alignments using the MView function in EMBL-EBI (Madeira *et al*. 2019). Workflow of methods can be seen in Supplementary Fig. 1.

## Results and discussion

### Protein-coding and noncoding regions exhibit differing levels of sequence conservation within *A. fumigatus*

To analyze the sequence diversity of noncoding regions across *A. fumigatus* strains (Fig. 2), we first identified 5,812 single-copy orthologous genes among 263 *A. fumigatus* strains and 2 *A. fischeri* strains. We then measured evolutionary conservation at individual alignment sites for protein-coding and noncoding regions across the 5,812 single-copy orthologous genes of interest. Of the 5,812 single-copy orthologous genes, 5,646 were found to be alignable; thus, we focused our subsequent analyses around these genes.

**Fig. 3.**
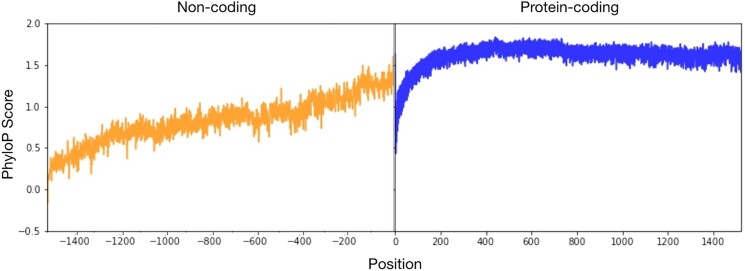
Noncoding regions are less conserved than protein-coding regions in the major fungal pathogen *A. fumigatus*. PhyloP score for protein-coding and noncoding regions. To determine conservation in protein-coding (blue / right panel) and noncoding (orange / left panel) regions, we calculated the PhyloP score across sites of all multiple nucleotide sequence alignments of protein-coding and upstream noncoding regions of 5,646 single-copy orthologs across 263 *A. fumigatus* strains and 2 *A. fischeri* strains. Scores of conservation were measured for individual nucleotide sites and scores were then averaged across all orthologs. Conserved sites have PhyloP scores above 0 and nonconserved sites have scores below 0. We find that in noncoding regions, sites that are closer to the start of the transcription start site (TSS) exhibit a higher level of conservation and generally decrease in conservation as we move further from the TSS. In protein-coding regions, PhyloP scores are generally above 0, which are indicative of high sequence conservation; the lowest scores are observed near the start of the protein-coding regions, which is likely an artifact caused by variation in starting codon position of gene annotations across *A. fumigatus* strains.

Examination of PhyloP scores for each protein-coding and noncoding alignment individually revealed that both protein-coding and noncoding regions exhibit varying levels of conservation across single-copy orthologous genes (Fig. 3). Examination of average PhyloP scores in protein-coding regions revealed a lower area of conservation near their start (first ∼100 bp). This may be due to slight differences in gene annotation between strains or the presence of genuine variation; utilization of alternate start sites for the same gene has been demonstrated in *Aspergillus* (Kjærbølling *et al*. 2020). Beyond the first ∼100 bp, conservation levels of protein-coding regions remain high throughout the first 1,500 bp. High conservation among protein-coding regions is also consistent with comparisons between *A. fumigatus* and closely related species (Fedorova *et al*. 2008).

The average PhyloP score in noncoding regions across *A. fumigatus* strains revealed that the highest levels of sequence conservation (as indicated by a higher PhyloP score) were directly upstream of the start site, with conservation generally decreasing further away from the start site. We also found that conservation begins to fade around 1,500 bp upstream of the start site (as indicated by a PhyloP score of 0). This pattern of higher sequence conservation in noncoding regions right upstream of the transcription start site is consistent with a previous study of noncoding regions comparing *A. fumigatus* and closely related species (Brown *et al*. 2022).

**Fig. 4.**
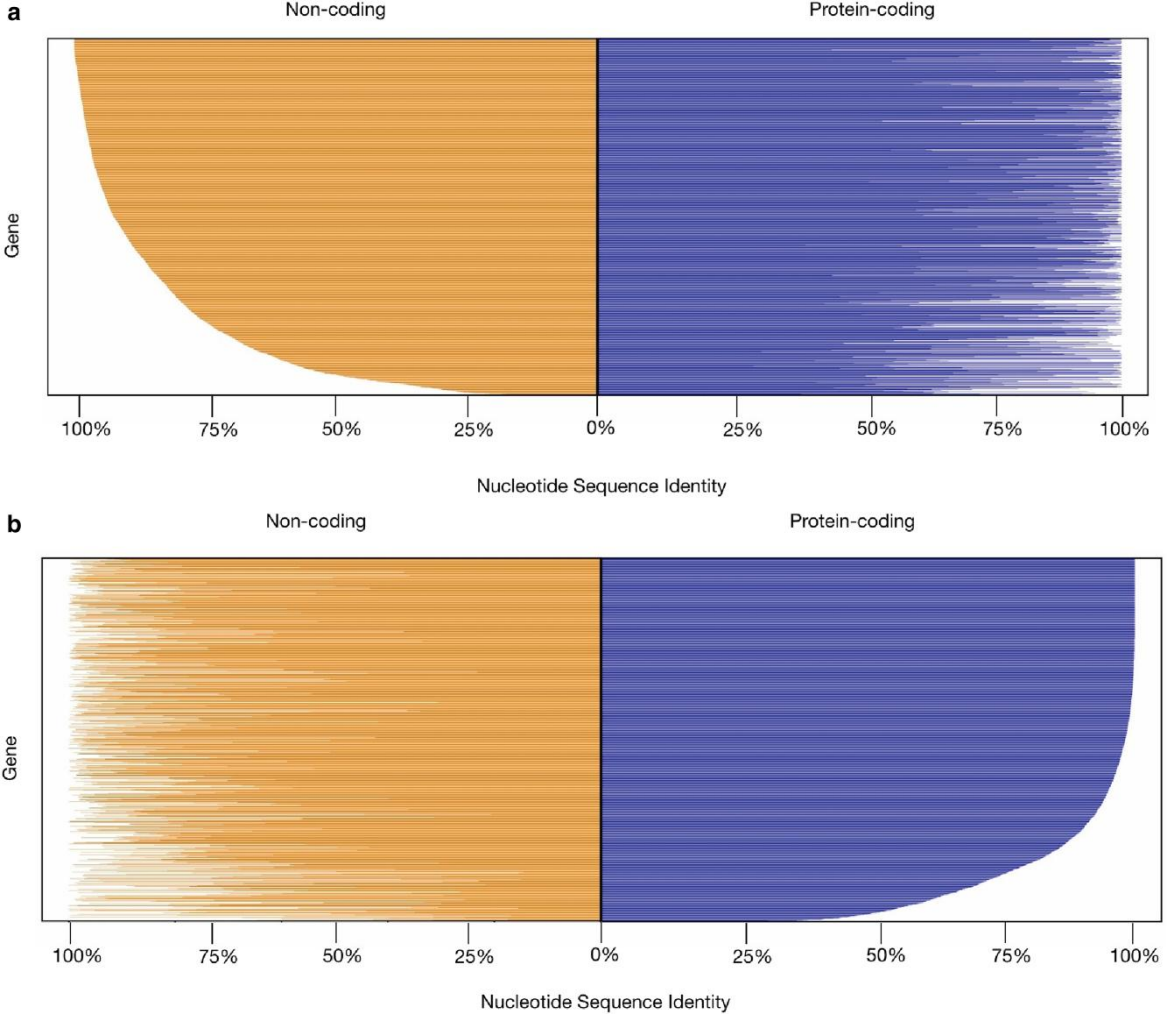
*A. fumigatus* orthologs exhibit numerous instances of highly conserved protein-coding genes whose noncoding regions are poorly conserved. Percent identity of protein-coding and noncoding regions of 5,646 *A. fumigatus* genes. Up to 1.5 kb upstream noncoding region (orange / left panels) is shown to the left were calculated and plotted by percent identity starting with 100% identity (top row) and descending. The associated protein-coding regions (blue / right panels) are shown to the right. Although the sequence conservation of protein-coding regions and the sequence conservation of their corresponding noncoding regions are correlated, there are numerous instances of genes with high protein-coding sequence identity and a lower identity in their noncoding region. a) *A. fumigatus* genes ranked by percent nucleotide sequence identity of their noncoding regions. b) *A. fumigatus* genes ranked by percent nucleotide sequence identity of their protein-coding regions.

To further examine the conservation of pairs of noncoding and protein-coding sequences, we calculated the percent identity for all single-copy orthologs in their protein-coding and associated noncoding regions (Fig. 4). We found that the percent nucleotide sequence identity of protein-coding regions exhibited a significant correlation (*r*^2^ = 0.143 and *P*-value <0.0001) with the percent nucleotide sequence identity of the associated noncoding regions (i.e. orthologs with higher percent identity in their protein-coding regions also exhibited higher percent identity in their noncoding regions). In a few cases, we also identified highly divergent protein-coding regions that were associated with highly similar noncoding regions.

We next focused on the 5,646 single-copy orthologs whose protein-coding sequences were alignable. Averaging the noncoding region percent identities for the 5,646 single-copy orthologous genes revealed an average identity of ∼85%, while the average protein-coding percent identity was ∼92%. Additionally, we found that 762 protein-coding alignments exhibited <75% nucleotide sequence identity, 2,482 exhibited sequence identity between ≥75 and <99%, and 2,402 exhibited ≥99% identity. For noncoding region alignments, 1,274 noncoding alignments exhibited <75% identity, 3,721 that exhibited sequence identity between ≥75 and <99%, and 817 that exhibited ≥99% identity. A 75% sequence identity cutoff was enforced because alignments with sequences that have percent identity values below 70–75% were often poor in our experience (e.g. one or more sequences would be often obviously misaligned). Given that alignments of high quality are required for the MK and HKA tests, we adopted the conservative cutoff of 75% sequence identity. Additionally, some level of polymorphisms and divergence is also required for these tests, so we also excluded sequences that exhibited sequence identities >99%.

### Many noncoding regions have signatures of positive selection

**Fig. 5.**
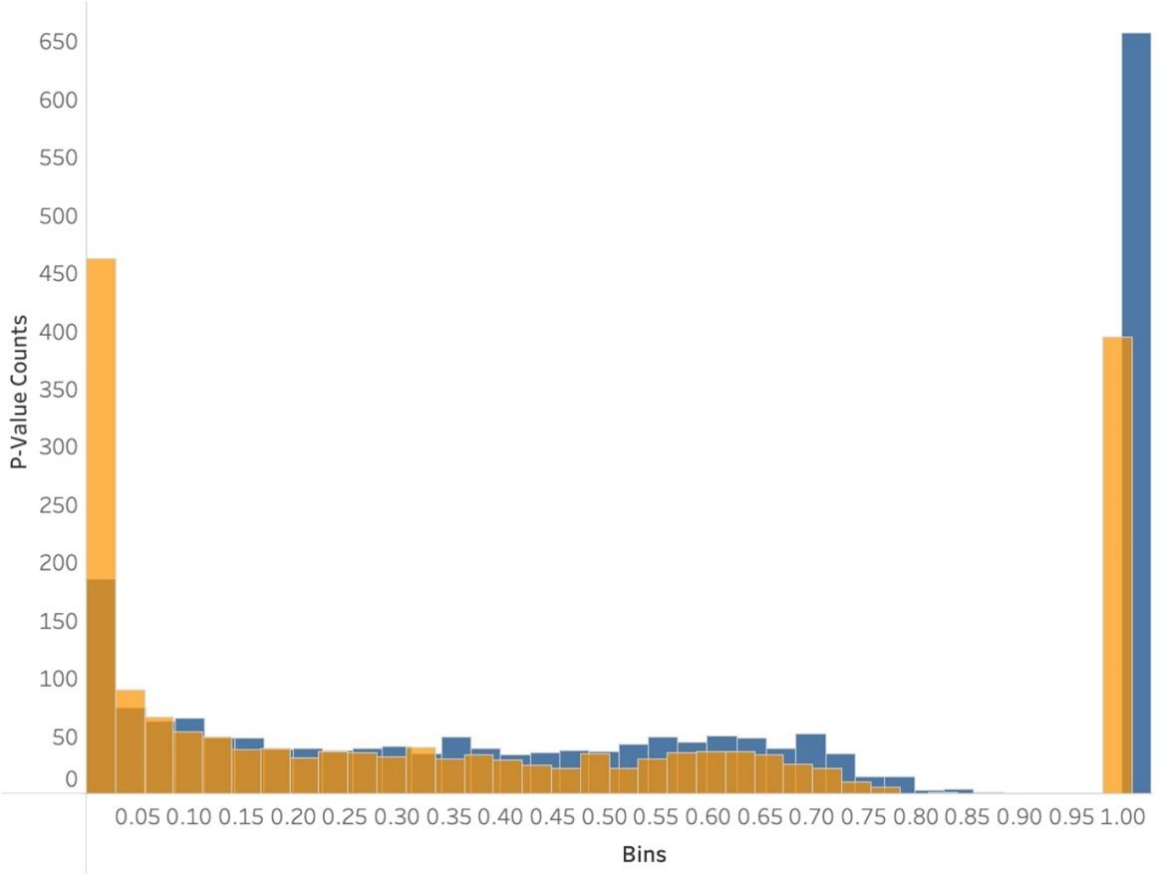
A higher number of noncoding regions than protein-coding regions exhibit signatures of selection under the MK test. Histogram of the distribution of *P*-values of the MK test. The MK test was calculated for 2,482 single-copy orthologs in both protein-coding (blue / darker) and noncoding regions (orange / lighter). Two hundred and seventeen protein-coding and 472 noncoding regions were found to be significant (*P* < 0.05).

To examine signatures of selection in *A. fumigatus* genes, we performed the MK and HKA tests of selection in 2,482 pairs of protein-coding and noncoding region alignments having sequence identity between ≥75 and <99%. For the MK test, we found that a total of 472/2,482 (19.0%) genes exhibited signatures of selection in their noncoding regions but not in their protein-coding regions, a total of 217/2,482 (8.7%) genes experienced selection in their protein-coding regions but not in their noncoding regions, and 144/2,482 (5.80%) genes experienced selection in both their protein-coding and noncoding regions (Fig. 5).

**Fig. 6.**
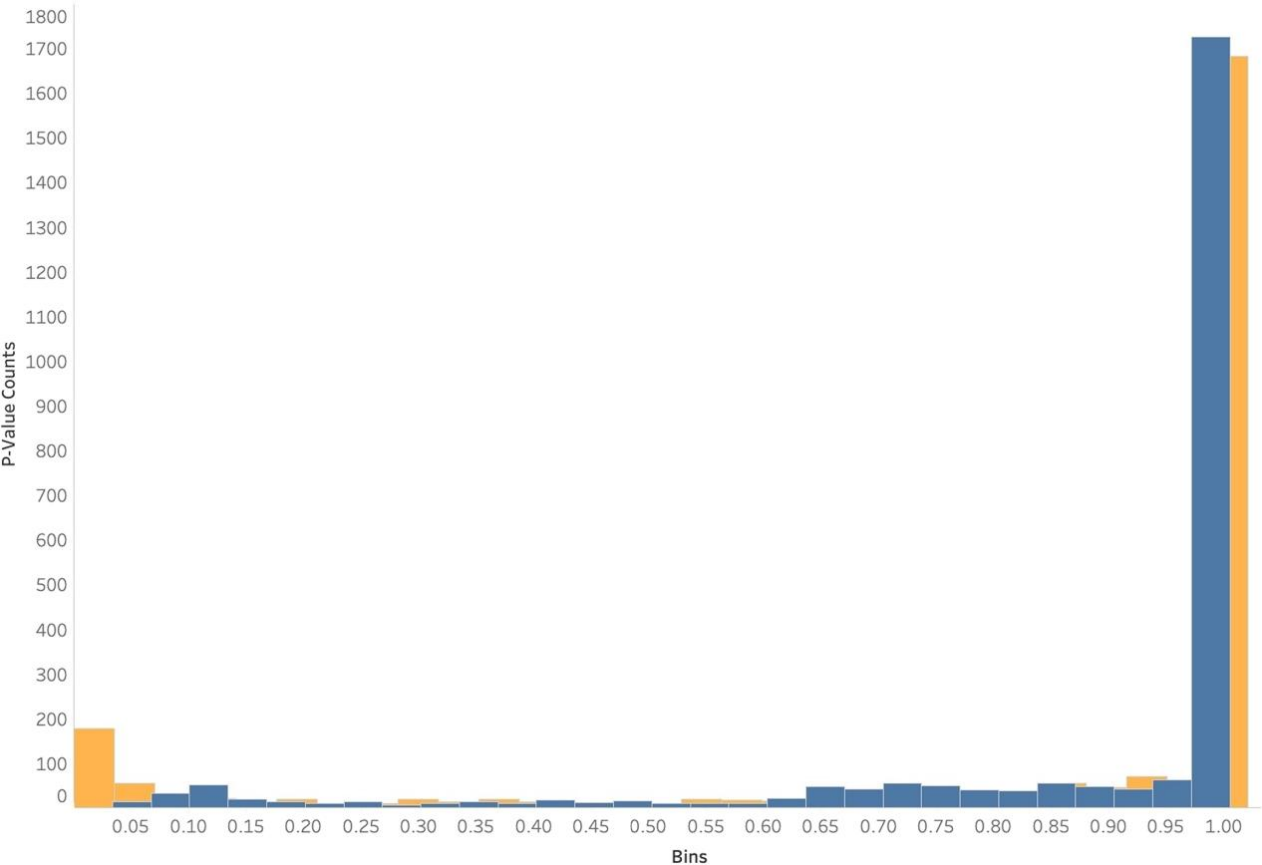
HKA test identified 207 noncoding and 4 protein-coding regions that exhibit a signature of selection. Histogram of the distribution of *P*-values of the HKA test across protein-coding (blue / darker) and noncoding (orange / lighter) regions of 2,482 genes. Two hundred and seven noncoding and 4 protein-coding regions were found to be significant (*P* < 0.05).

**Fig. 7.**
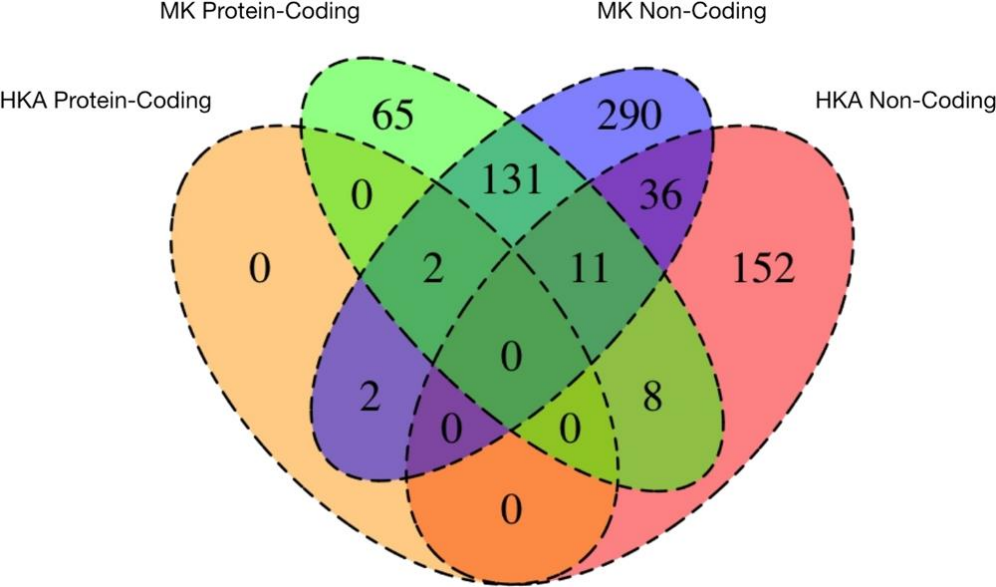
Venn diagram of significant results from HKA protein-coding, MK-protein-coding, MK noncoding, and HKA noncoding tests. There were 478 (290 + 36 + 152) genes with evidence of selection only in their noncoding regions compared with 65 genes with evidence of selection only in their protein-coding regions across both MK and HKA tests.

For the HKA test, we found 4/2,482 (8.8%) and 207/2,482 genes with evidence of positive selection in their protein-coding and noncoding regions, respectively (Fig. 6). Examination of the genes that were significant under the MK and HKA tests shows that there is relatively limited overlap for both protein-coding and noncoding regions (Fig. 7). For example, only 36 noncoding regions exclusively exhibit evidence of selection by both tests. For protein-coding regions, the lack of overlap is largely due to the very small number of protein-coding regions that show evidence of selection in the HKA test. For the noncoding regions, the limited overlap is likely due to the differences in the neutral sites used by the 2 tests (the MK test uses the synonymous sites of the corresponding protein-coding region, whereas the HKA test uses all the sites of a neutrally evolving noncoding region).

### Genes with evidence of selection in noncoding regions are enriched for binding and regulatory activity, including 21 genes involved in *A. fumigatus* virulence

We used GO enrichment to determine whether any functions were overrepresented. For the 472 genes with evidence of selection under the MK test in their noncoding regions, we found 22 categories that were enriched for molecular function. “DNA binding” was the top term identified for molecular function (*P* = 9.06 × 10^−8^) and the most enriched term overall. “Cytoskeleton motor activity” (*P* = 6.59 × 10^−6^), “ion binding” (*P* = 7.33 × 10^−5^), and “transcription factor binding” (*P* = 4.08 × 10^−4^) were also represented grouped terms for molecular function components, respectively (Supplementary Table 7). For the 217 genes with evidence of selection in their protein-coding regions, 7 molecular functions were overrepresented, including “ATP hydrolysis activity” (*P* = 1.37 × 10^−3^) and various functions involved in binding activities. For the HKA test, we find that enzyme regulator activity (*P* = 3.33 × 10^−2^) was the only molecular term found for the noncoding regions, and no GO terms were enriched for the HKA protein-coding results (Supplementary Table 7). Additionally, the 36 genes that experienced selection in their noncoding regions under both the HKA and MK tests are enriched for various regulatory processes (Supplementary Table 7).

We compared our list of genes under selection to a previously curated set of 206 genetic determinants of *A. fumigatus* virulence (Steenwyk *et al*. 2021). Given that *A. fumigatus* strains have been demonstrated to exhibit differences in virulence in mouse models of fungal disease (Keizer *et al*. 2021), selection in the noncoding or protein-coding regions of these genes may be relevant to *A. fumigatus* virulence. We found that the noncoding regions of 18 of the 206 virulence genes were under selection according to the MK test (*argEF*, *ags1*, *csmB*, *pabA*, *medA*, *mtfA*, *myoB*, *myoE*, *pld2*, g*liP*, *rgsC*, *aceA*, *atfA*, *cch1*, *fbx15*, *flcB*, *schA*, and *zrfB*) and 3 according to the HKA test (*cds1*, *nop4*, and *dvrA*; Supplementary Table 8). We found that the most represented general function among these 18 genes was “stress response,” which raises the question of their impact on virulence, given the role that stress response has been shown to play in *A. fumigatus* virulence (Colabardini *et al*. 2022); for example, exposure to different temperatures, pH, and drug treatments leads to differences in gene expression (Latgé and Chamilos 2019; Colabardini *et al*. 2022). We also identified 4 protein-coding regions of virulence genes (*lysF*, *myoB*, *aftA*, and *fbx15*) that showed evidence of selection under the MK test. Similarly, we found 11 virulence genes (*hcsA*, *lysF*, *ags1*, *chsG*, *erg12*, *rtfA*, *tom24*, *fmaE*, *gliC*, *gliT*, and *sidI*) with evidence of selection in their noncoding regions under the HKA test, and 1 (*aceA*) with evidence of selection in its protein-coding region (Supplementary Table 8).

### Examples of noncoding region differences between *A. fumigatus* strains

**Fig. 8.**
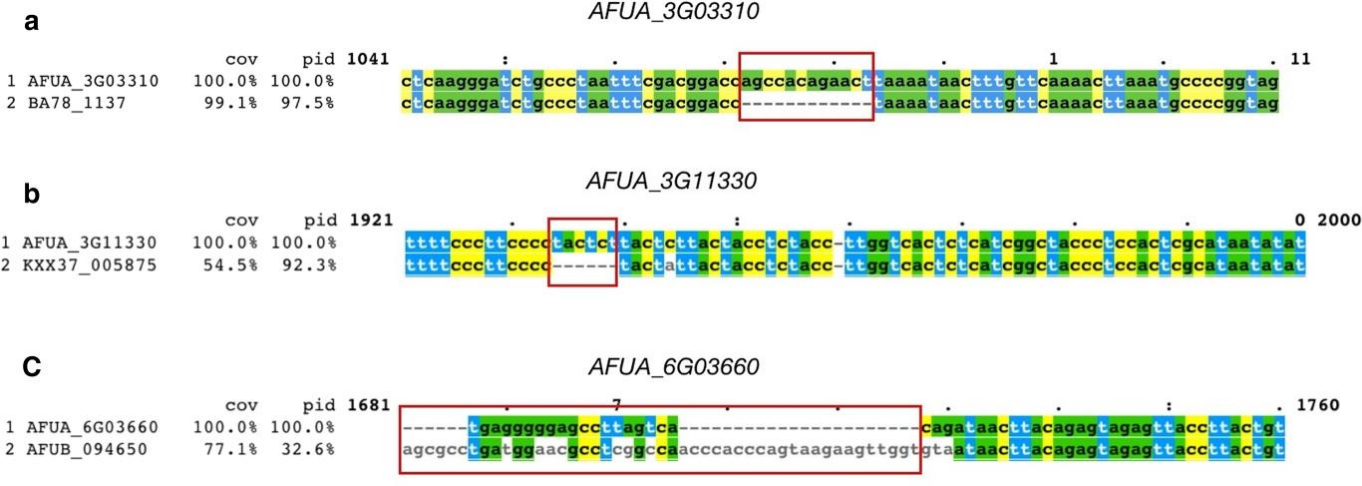
Notable examples of sequence differences between *A. fumigatus* strains in noncoding regions. Here, we show 3 regions of sequence alignments of noncoding regions that differ between *A. fumigatus* Af293 and another *A. fumigatus* strain. a) AFUA_3G03310 (RTA1 domain protein) exhibits a 12-bp region present in *A. fumigatus* Af293 but absent in several other strains, including BA78_1337. b) AFUA_3G11330 (transcription factor AtfA) exhibits a 6-bp region absent in *A. fumigatus* Af293 but present in several other strains, including KXX37_005875. c) AFUA_6G03660 (predicted to be involved in the production of biotin) exhibits a noncoding difference between the 2 reference strains of *A. fumigatus* (Af293 and A1163).

We next sought to identify representative sequence differences in noncoding regions between *A. fumigatus* strains that exhibited signatures of selection according to the MK test or the HKA test (Fig. 8) based on the nucleotide variation in the region. One such example was the noncoding region of *AFUA_3G03310*, which is under selection according to the MK test. The noncoding region exhibits a 12-bp region (AGCCACAGAACT) present in *A. fumigatus* Af293 and 91 other strains but absent from the rest. This binding-site location is an exact match to the Met31 transcription factor–binding site involved in sulfur metabolism in *S. cerevisiae* (Cormier *et al*. 2010). The MetZ transcription factor performs a similar function in *Aspergillus nidulans* (Pilsyk *et al*. 2015) and may also be in *A. fumigatus* (Amich *et al*. 2016). Another gene with evidence of selection in its noncoding region is *AFUA_3G11330*, which encodes the putative transcription factor AftA involved in stress response and spore viability in *Aspergillus* (Lara-Rojas *et al*. 2011). The *AFUA_3G11330* noncoding region exhibits a 6-bp region (TACTCT) present in *A. fumigatus* Af293 and about half of the other *A. fumigatus* strains while absent in the other *A. fumigatus* strains. This 6 bp region is similar to the 6 bp binding site for Yap1 in *S. cerevisiae*, which is required for oxidative stress tolerance (Natkańska *et al*. 2017). An ortholog of Yap1 is known to be involved in voriconazole resistance in *A*spergillus *flavus* (Ukai *et al*. 2018) and may play a role in stress response in *A. fumigatus*, which is important as a mechanism for survival within the human lung (Latgé and Chamilos 2019). Both the MK and HKA tests found signatures of selection in *AFUA_6G03660*, an uncharacterized gene in *A. fumigatus*. Although of unknown function, this gene is of particular interest as its noncoding region differs between the 2 reference strains *A. fumigatus* Af293 and *A. fumigatus* A1163, which vary in their virulence in animal models of fungal disease (Colabardini *et al*. 2022). *A. fumigatus* A1163 exhibits a larger region of 22 bp that is absent in *A. fumigatus* A1163. This region helps to illustrate the complex noncoding sequence differences between *A. fumigatus* strains, including those that are closely related.

## Conclusion

Here, we presented a comprehensive study of signatures of positive selection in both protein-coding and noncoding regions across many strains of the fungal pathogen *A. fumigatus*. We identified several noncoding regions under selection in *A. fumigatus*, including several candidate transcription factor–binding sites that differ between strains are await further exploration. While some of our selection tests, such the MK test, revealed that a considerable fraction (∼20%) of the noncoding regions tested exhibited signatures of selection, it is worth noting that our methods filtered out certain groups of genes (e.g. the 2,402 regions that were >99% identical were not tested). Thus, it is hard to infer from our data the actual percentages of noncoding and protein-coding regions that are under selection in the *A. fumigatus* genome. Furthermore, the genome of *A. fumigatus* contains ∼10,000 protein-coding genes; thus, it is more appropriate to infer that ∼5% of genes display signatures of selection in their noncoding regions.

More broadly, these findings suggest divergence in noncoding sequences may play an important role in population variation among fungal pathogens and beyond. Currently, there are no datasets available that report genome-wide differential expression data for *A. fumigatus* strains. Experiments that examine the differential expression of genes in diverse *A. fumigatus* strains or the functional implications of the noncoding region differences we have identified will be of great interest.

## Supplementary Material

jkae091_Supplementary_Data

## Data Availability

All *Aspergillus* genomes are publicly available and were downloaded from NCBI (https://www.ncbi.nlm.nih.gov/) and are catalogued in Supplementary Table 1. The custom Python script for retrieval of noncoding sequences can be found at https://github.com/alecbrown24/General_Bio_Scripts (adapted from https://github.com/shenwei356/bio_scripts). Supplemental material available at G3 online.

## References

[jkae091-B1] Amich J, Dümig M, O’Keeffe G, Binder J, Doyle S, Beilhack A, Krappmann S. 2016. Exploration of sulfur assimilation of *Aspergillus fumigatus* reveals biosynthesis of sulfur-containing amino acids as a virulence determinant. Infect Immun. 84(4):917–929. doi:10.1128/IAI.01124-15. 26787716 PMC4807484

[jkae091-B2] Barber AE, Sae-Ong T, Kang K, Seelbinder B, Li J, Walther G, Panagiotou G, Kurzai O. 2021. *Aspergillus fumigatus* pan-genome analysis identifies genetic variants associated with human infection. Nat Microbiol. 6(12):1526–1536. doi:10.1038/s41564-021-00993-x. 34819642

[jkae091-B3] Bertuzzi M, Hayes GE, Icheoku UJ, van Rhijn N, Denning DW, Osherov N, Bignell E. 2018. Anti-Aspergillus activities of the respiratory epithelium in health and disease. J Fungi (Basel). 4(1):8. doi:10.3390/jof4010008. 29371501 PMC5872311

[jkae091-B4] Bhabhra R, Askew DS. 2005. Thermotolerance and virulence of *Aspergillus fumigatus*: role of the fungal nucleolus. Med Mycol. 43(Suppl 1):87–93. doi:10.1080/13693780400029486. 16110798

[jkae091-B5] Bongomin F, Gago S, Oladele RO, Denning DW. 2017. Global and multi-national prevalence of fungal diseases—estimate precision. J Fungi. 3(4):57. doi:10.3390/jof3040057. PMC5753159 29371573

[jkae091-B6] Brown A, Mead ME, Steenwyk JL, Goldman GH, Rokas A. 2022. Extensive non-coding sequence divergence between the major human pathogen *Aspergillus fumigatus* and its relatives. Front Fungal Biol. 3:802494. doi:10.3389/ffunb.2022.802494. 36866034 PMC9977105

[jkae091-B7] Buchfink B, Xie C, Huson DH. 2015. Fast and sensitive protein alignment using DIAMOND. Nat Methods. 12(1):59–60. doi:10.1038/nmeth.3176. 25402007

[jkae091-B8] Cadena J, Thompson GR, Patterson TF. 2021. Aspergillosis: epidemiology, diagnosis, and treatment. Infect Dis Clin North Am. 35(2):415–434. doi:10.1016/j.idc.2021.03.008. 34016284

[jkae091-B9] Carroll SB . 2005. Evolution at two levels: on genes and form. PLoS Biol. 3(7):e245. doi:10.1371/journal.pbio.0030245. 16000021 PMC1174822

[jkae091-B10] Chotirmall SH, Al-Alawi M, Mirkovic B, Lavelle G, Logan PM, Greene CM, McElvaney NG. 2013. Aspergillus-associated airway disease, inflammation, and the innate immune response. Biomed Res Int. 2013:723129. doi:10.1155/2013/723129. 23971044 PMC3736487

[jkae091-B11] Colabardini AC, Wang F, Dong Z, Pardeshi L, Rocha MC, Costa JH, dos Reis TF, Brown A, Jaber QZ, Fridman M, et al 2022. Heterogeneity in the transcriptional response of the human pathogen *Aspergillus fumigatus* to the antifungal agent caspofungin. Genetics. 220(1):iyab183. doi:10.1093/genetics/iyab183. 34718550 PMC8733440

[jkae091-B12] Cormier L, Barbey R, Kuras L. 2010. Transcriptional plasticity through differential assembly of a multiprotein activation complex. Nucleic Acids Res. 38(15):4998–5014. doi:10.1093/nar/gkq257. 20392822 PMC2926612

[jkae091-B13] Croft CA, Culibrk L, Moore MM, Tebbutt SJ. 2016. Interactions of *Aspergillus fumigatus* conidia with airway epithelial cells: a critical review. Front Microbiol. 7:472. doi:10.3389/fmicb.2016.00472. 27092126 PMC4823921

[jkae091-B14] Denning DW . 2024. Global incidence and mortality of severe fungal disease. Lancet Infect Dis. S1473-3099(23)00692-8. [Online ahead of print]. doi:10.1016/S1473-3099(23)00692-8. 38224705

[jkae091-B15] de Vries RP, Riley R, Wiebenga A, Aguilar-Osorio G, Amillis S, Uchima CA, Anderluh G, Asadollahi M, Askin M, Barry K, et al 2017. Comparative genomics reveals high biological diversity and specific adaptations in the industrially and medically important fungal genus *Aspergillus*. Genome Biol. 18(1):28. doi:10.1186/s13059-017-1151-0. 28196534 PMC5307856

[jkae091-B16] Elyashiv E, Bullaughey K, Sattath S, Rinott Y, Przeworski M, Sella G. 2010. Shifts in the intensity of purifying selection: an analysis of genome-wide polymorphism data from two closely related yeast species. Genome Res. 20(11):1558–1573. doi:10.1101/gr.108993.110. 20817943 PMC2963819

[jkae091-B17] Emms DM, Kelly S. 2015. OrthoFinder: solving fundamental biases in whole genome comparisons dramatically improves orthogroup inference accuracy. Genome Biol. 16(1):157. doi:10.1186/s13059-015-0721-2. 26243257 PMC4531804

[jkae091-B18] Fedorova ND, Khaldi N, Joardar VS, Maiti R, Amedeo P, Anderson MJ, Crabtree J, Silva JC, Badger JH, Albarraq A, et al 2008. Genomic islands in the pathogenic filamentous fungus *Aspergillus fumigatus*. PLoS Genet. 4(4):e1000046. doi:10.1371/journal.pgen.1000046. 18404212 PMC2289846

[jkae091-B19] Ferretti L, Raineri E, Ramos-Onsins S. 2012. Neutrality tests for sequences with missing data. Genetics. 191(4):1397–1401. doi:10.1534/genetics.112.139949. 22661328 PMC3416018

[jkae091-B20] Flores MEB, Medina PG, Camacho SPD, de Jesús Uribe Beltrán M, del Carmen De la Cruz Otero M, Ramírez IO, Ernesto Tiznado Hernández M. 2014. Fungal spore concentrations in indoor and outdoor air in university libraries, and their variations in response to changes in meteorological variables. Int J Environ Health Res. 24(4):320–340. doi:10.1080/09603123.2013.835029. 24070332

[jkae091-B21] Hill MS, Vande Zande P, Wittkopp PJ. 2021. Molecular and evolutionary processes generating variation in gene expression. Nat Rev Genet. 22(4):203–215. doi:10.1038/s41576-020-00304-w. 33268840 PMC7981258

[jkae091-B22] Horta MAC, Steenwyk JL, Mead ME, Dos Santos LHB, Zhao S, Gibbons JG, Marcet-Houben M, Gabaldón T, Rokas A, Goldman GH. 2022. Examination of genome-wide ortholog variation in clinical and environmental isolates of the fungal pathogen *Aspergillus fumigatus*. mBio. 13(4):e0151922. doi:10.1128/mbio.01519-22. 35766381 PMC9426589

[jkae091-B23] Hudson RR, Kreitman M, Aguadé M. 1987. A test of neutral molecular evolution based on nucleotide data. Genetics. 116(1):153–159. doi:10.1093/genetics/116.1.153. 3110004 PMC1203113

[jkae091-B24] Katoh K, Misawa K, Kuma K, Miyata T. 2002. MAFFT: a novel method for rapid multiple sequence alignment based on fast Fourier transform. Nucleic Acids Res. 30:3059–3066. doi:10.1093/nar/gkf436. 12136088 PMC135756

[jkae091-B25] Keizer EM, Valdes ID, Forn-Cuni G, Klijn E, Meijer AH, Hillman F, Wösten HAB, de Cock H. 2021. Variation of virulence of five *Aspergillus fumigatus* isolates in four different infection models. PLoS One. 16(7):e0252948. doi:10.1371/journal.pone.0252948. 34242260 PMC8270121

[jkae091-B26] Kjærbølling I, Vesth T, Frisvad JC, Nybo JL, Theobald S, Kildgaard S, Petersen TI, Kuo A, Sato A, Lyhne EK, et al 2020. A comparative genomics study of 23 *Aspergillus* species from section Flavi. Nat Commun. 11(1):1106. doi:10.1038/s41467-019-14051-y. 32107379 PMC7046712

[jkae091-B27] Lara-Rojas F, Sánchez O, Kawasaki L, Aguirre J. 2011. *Aspergillus nidulans* transcription factor AtfA interacts with the MAPK SakA to regulate general stress responses, development and spore functions. Mol Microbiol. 80(2):436–454. doi:10.1111/j.1365-2958.2011.07581.x. 21320182 PMC3108070

[jkae091-B28] Latgé J-P, Chamilos G. 2019. *Aspergillus fumigatus* and Aspergillosis in 2019. Clin Microbiol Rev. 33(1):e00140-18. doi:10.1128/CMR.00140-18. 31722890 PMC6860006

[jkae091-B29] Lofgren LA, Ross BS, Cramer RA, Stajich JE. 2022. The pan-genome of *Aspergillus fumigatus* provides a high-resolution view of its population structure revealing high levels of lineage-specific diversity driven by recombination. PLoS Biol. 20(11):e3001890. doi:10.1371/journal.pbio.3001890. 36395320 PMC9714929

[jkae091-B30] Madeira F, Park YM, Lee J, Buso N, Gur T, Madhusoodanan N, Basutkar P, Tivey ARN, Potter SC, Finn RD, et al 2019. The EMBL-EBI search and sequence analysis tools APIs in 2019. Nucleic Acids Res. 47(W1):W636–W641. doi:10.1093/nar/gkz268. 30976793 PMC6602479

[jkae091-B31] McDonald JH, Kreitman M. 1991. Adaptive protein evolution at the Adh locus in *Drosophila*. Nature. 351(6328):652–654. doi:10.1038/351652a0. 1904993

[jkae091-B32] Mead ME, Knowles SL, Raja HA, Beattie SR, Kowalski CH, Steenwyk JL, Silva LP, Chiaratto J, Ries LNA, Goldman GH, et al 2019. Characterizing the pathogenic, genomic, and chemical traits of *Aspergillus fischeri*, a close relative of the major human fungal pathogen *Aspergillus fumigatus*. mSphere. 4(1):e00018–e00019. doi:10.1128/mSphere.00018-19. 30787113 PMC6382966

[jkae091-B33] Mead ME, Steenwyk JL, Silva LP, de Castro PA, Saeed N, Hillmann F, Goldman GH, Rokas A. 2021. An evolutionary genomic approach reveals both conserved and species-specific genetic elements related to human disease in closely related *Aspergillus* fungi. Genetics. 218(2):iyab066. doi:10.1093/genetics/iyab066. 33944921 PMC8225353

[jkae091-B34] Minh BQ, Schmidt HA, Chernomor O, Schrempf D, Woodhams MD, von Haeseler A, Lanfear R. 2020. IQ-TREE 2: new models and efficient methods for phylogenetic inference in the genomic era. Mol Biol Evol. 37(5):1530–1534. doi:10.1093/molbev/msaa015. 32011700 PMC7182206

[jkae091-B35] Murga-Moreno J, Coronado-Zamora M, Hervas S, Casillas S, Barbadilla A. 2019. iMKT: the integrative McDonald and Kreitman test. Nucleic Acids Res. 47(W1):W283–W288. doi:10.1093/nar/gkz372. 31081014 PMC6602517

[jkae091-B36] Natkańska U, Skoneczna A, Sieńko M, Skoneczny M. 2017. The budding yeast orthologue of Parkinson's disease-associated DJ-1 is a multi-stress response protein protecting cells against toxic glycolytic products. Biochim Biophys Acta Mol Cell Res. 1864(1):39–50. doi:10.1016/j.bbamcr.2016.10.016. 27984092

[jkae091-B37] Park H-S, Yu J-H. 2016. Developmental regulators in *Aspergillus fumigatus*. J Microbiol. 54(3):223–231. doi:10.1007/s12275-016-5619-5. 26920882

[jkae091-B38] Piłsyk S, Natorff R, Sieńko M, Skoneczny M, Paszewski A, Brzywczy J. 2015. The *Aspergillus nidulans* metZ gene encodes a transcription factor involved in regulation of sulfur metabolism in this fungus and other Eurotiales. Curr Genet. 61(2):115–125. doi:10.1007/s00294-014-0459-5. 25391366

[jkae091-B39] Ramani R, Krumholz K, Huang Y-F, Siepel A. 2019. PhastWeb: a web interface for evolutionary conservation scoring of multiple sequence alignments using phastCons and phyloP. Bioinformatics. 35(13):2320–2322. doi:10.1093/bioinformatics/bty966. 30481262 PMC6596881

[jkae091-B40] Raudvere U, Kolberg L, Kuzmin I, Arak T, Adler P, Peterson H, Vilo J. 2019. G:Profiler: a web server for functional enrichment analysis and conversions of gene lists (2019 update). Nucleic Acids Res. 47(W1):W191–W198. doi:10.1093/nar/gkz369. 31066453 PMC6602461

[jkae091-B41] Rees CA, Stefanuto P-H, Beattie SR, Bultman KM, Cramer RA, Hill JE. 2017. Sniffing out the hypoxia volatile metabolic signature of *Aspergillus fumigatus*. J Breath Res. 11(3):036003. doi:10.1088/1752-7163/aa7b3e. 28825403 PMC5793853

[jkae091-B42] Rokas A, Mead ME, Steenwyk JL, Oberlies NH, Goldman GH. 2020. Evolving moldy murderers: Aspergillus section Fumigati as a model for studying the repeated evolution of fungal pathogenicity. PLoS Pathog. 16(2):e1008315. doi:10.1371/journal.ppat.1008315. 32106242 PMC7046185

[jkae091-B43] Steenwyk JL, Balamurugan C, Raja HA, Gonçalves C, Li N, Martin F, Berman J, Oberlies NH, Gibbons JG, Goldman GH, et al 2024. Phylogenomics reveals extensive misidentification of fungal strains from the genus *Aspergillus*. Microbiol Spectr. 12(4):e0398023. doi:10.1128/spectrum.03980-23. 38445873 PMC10986620

[jkae091-B44] Steenwyk JL, Mead ME, de Castro PA, Valero C, Damasio A, dos Santos RAC, Labella AL, Li Y, Knowles SL, Raja HA, et al 2021. Genomic and phenotypic analysis of COVID-19-associated pulmonary Aspergillosis isolates of *Aspergillus fumigatus*. Microbiol Spectr. 9(1):e0001021. doi:10.1128/Spectrum.00010-21. 34106569 PMC8552514

[jkae091-B45] Steenwyk JL, Mead ME, Knowles SL, Raja HA, Roberts CD, Bader O, Houbraken J, Goldman GH, Oberlies NH, Rokas A, 2020. Variation among biosynthetic gene clusters, secondary metabolite profiles, and cards of virulence across *Aspergillus* species. Genetics. 216(2):481–497. doi:10.1534/genetics.120.303549. 32817009 PMC7536862

[jkae091-B449] Steenwyk JL, Rokas A. 2019. Treehouse: a user-friendly application to obtain subtrees from large phylogenies. BMC Res Notes. 12(1):541. doi:10.1186/s13104-019-4577-5. 31455362 PMC6712805

[jkae091-B46] Steinbach WJ, Marr KA, Anaissie EJ, Azie N, Quan S-P, Meier-Kriesche H-U, Apewokin S, Horn DL. 2012. Clinical epidemiology of 960 patients with invasive *Aspergillosis* from the PATH alliance registry. J Infect. 65(5):453–464. doi:10.1016/j.jinf.2012.08.003. 22898389

[jkae091-B47] Suyama M, Torrents D, Bork P. 2006. PAL2NAL: robust conversion of protein sequence alignments into the corresponding codon alignments. Nucleic Acids Res. 34(Web Server):W609–W612. doi:10.1093/nar/gkl315. 16845082 PMC1538804

[jkae091-B48] Ukai Y, Kuroiwa M, Kurihara N, Naruse H, Homma T, Maki H, Naito A. 2018. Contributions of yap1 mutation and subsequent atrF upregulation to voriconazole resistance in *Aspergillus flavus*. Antimicrob Agents Chemother. 62(11):e01216-18. doi:10.1128/AAC.01216-18. 30126960 PMC6201102

[jkae091-B49] Vinet L, Zhedanov A. 2011. A ‘missing’ family of classical orthogonal polynomials. J Phys A: Math Theor. 44(8):085201. doi:10.1088/1751-8113/44/8/085201.

[jkae091-B50] Waddell PJ, Steel MA. 1997. General time-reversible distances with unequal rates across sites: mixing gamma and inverse Gaussian distributions with invariant sites. Mol Phylogenet Evol. 8(3):398–414. doi:10.1006/mpev.1997.0452. 9417897

[jkae091-B51] WHO . 2022. WHO releases First-Ever List of Health-Threatening Fungi. Genava: WHO. Available at: https://www.who.int/news/item/25-10-2022-who-releases-first-ever-list-of-health-threatening-fungi [accessed2023 Sept 8]. PMC10043918 36379529

[jkae091-B52] Wirmann L, Ross B, Reimann O, Steinmann J, Rath P-M. 2018. Airborne *Aspergillus fumigatus* spore concentration during demolition of a building on a hospital site, and patient risk determination for invasive Aspergillosis including azole resistance. J Hosp Infect. 100(3):e91–e97. doi:10.1016/j.jhin.2018.07.030. 30056016

[jkae091-B53] Wong TKF, Kalyaanamoorthy S, Meusemann K, Yeates DK, Misof B, Jermiin LS. 2020. A minimum reporting standard for multiple sequence alignments. NAR Genom Bioinform. 2(2):lqaa024. doi:10.1093/nargab/lqaa024. 33575581 PMC7671350

[jkae091-B54] Xue KS, Bloom JD. 2020. Linking influenza virus evolution within and between human hosts. Virus Evol. 6(1):veaa010. doi:10.1093/ve/veaa010. 32082616 PMC7025719

