# Supplementary figures and images for: Genome-wide patterns of noncoding and protein-coding sequence variation in the major fungal pathogen Aspergillus fumigatus
Source: G3 (Bethesda). 2024 May 2;14(7):jkae091. doi: 10.1093/g3journal/jkae091 (PMC11228837; doi:10.1093/g3journal/jkae091)

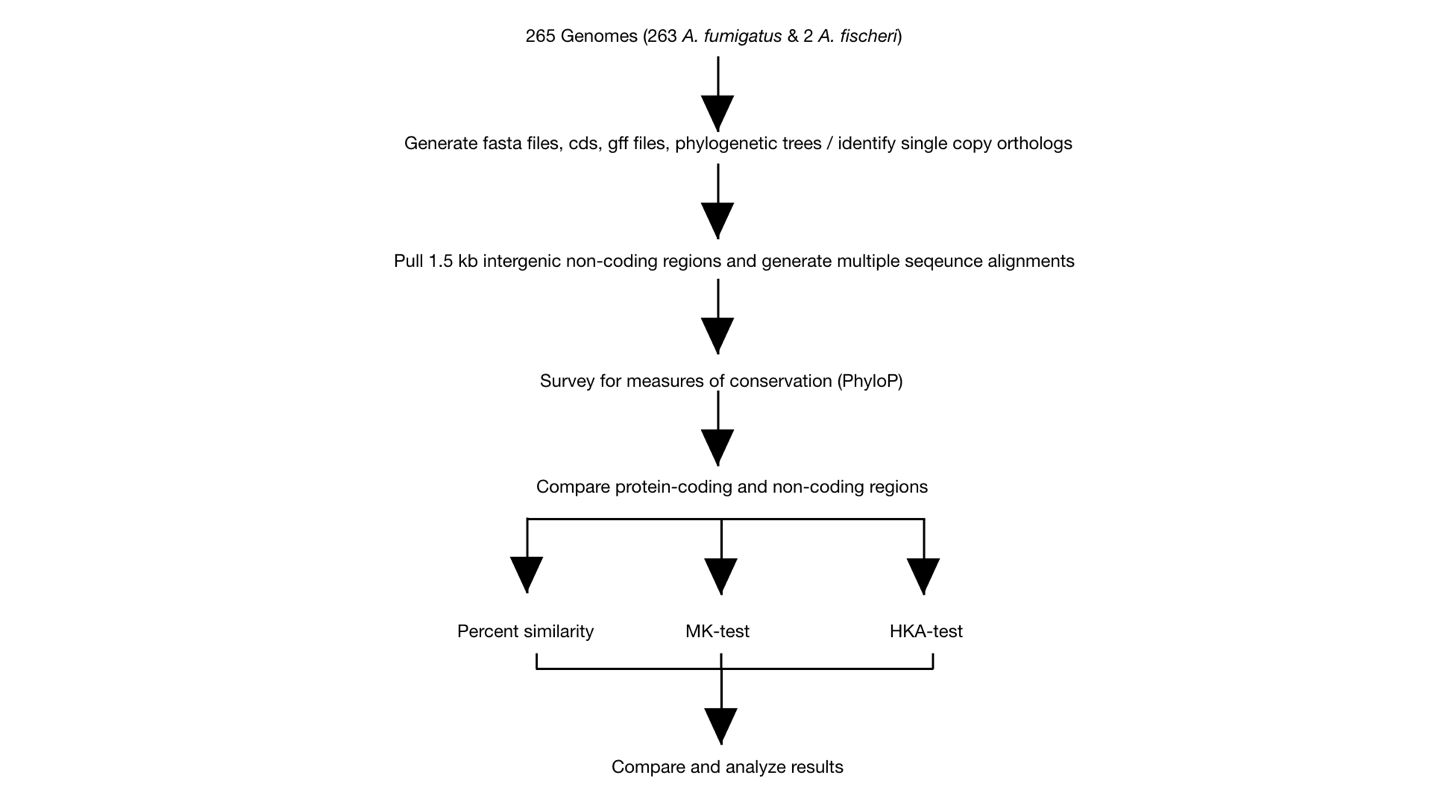


**Figure S1. Flow chart of methods used in this study.**

Supplement: jkae091_Supplementary_Data [file jkae091_supplementary_data.zip › Figure_S1_G3-2023-404800.docx]
